# Probable Causative Agents and Demographic Patterns of Encephalitis, Meningitis, and Meningoencephalitis in a Single Tertiary Care Center

**DOI:** 10.7759/cureus.68707

**Published:** 2024-09-05

**Authors:** Ahmed Attar, Abdulrahman M Khojah, Abdulrazak M Sakhakhni, Hussam Alasmari, Abdulaziz Bamusa, Yousef Alharbi, Talal Alajmi, Mohamed E Ahmed, Abdullah A Awadh

**Affiliations:** 1 Department of Neurosciences, Ministry of the National Guard - Health Affairs, Jeddah, SAU; 2 Research Office, King Abdullah International Medical Research Center, Jeddah, SAU; 3 College of Medicine, King Saud Bin Abdulaziz University for Health Sciences, Jeddah, SAU; 4 Department of Medicine, McMaster University, Hamilton, CAN; 5 College of Medicine, King Saud Bin Abdulaziz University for Health Sciences, Riyadh, SAU; 6 Department of Critical Care Medicine, Ministry of the National Guard - Health Affairs, Jeddah, SAU; 7 Department of Biostatistics, King Abdullah International Medical Research Center, Jeddah, SAU; 8 College of Basic Medical Science, King Saud Bin Abdulaziz University for Health Sciences, Jeddah, SAU

**Keywords:** acute encephalitis, bacterial cns infection, complicated meningitis, saudi population, viral cns infection

## Abstract

Introduction

Encephalitis, meningitis, and meningoencephalitis present significant challenges in clinical management owing to their diverse etiologies and potential complications. A high suspicion index is critical for guiding treatment strategies and improving patient outcomes. Understanding the demographic characteristics and frequency of causes of these conditions is essential to deliver optimized care.

Objective

This study aimed to investigate epidemiological causes and relative outcomes, including mortality, based on cultures, laboratory investigations, and demographic factors among patients with encephalitis, meningitis, and meningoencephalitis in a Saudi Arabian tertiary care center.

Methods

A retrospective cross-sectional study was conducted at King Abdulaziz Medical City (KAMC) in Jeddah, Saudi Arabia. Data were collected from patients admitted between April 2016 and December 2022 who met the specified inclusion criteria.

Results

Among 233 patients, meningitis was the most prevalent diagnosis (65.77%), with bacterial agents being the predominant causative agents (79.74%). Higher mortality was significant with pediatrics <5 years and adults >60 years.

Conclusion

This study provides valuable insights into the epidemiology and clinical outcomes of central neurological infections based on a Saudi Arabian cohort. These findings underscore the importance of an accurate diagnosis and tailored management strategies. Further studies are warranted to enhance our understanding and to inform more predictable characteristics targeted in optimizing healthcare delivery for patients with such conditions.

## Introduction

Encephalitis, meningitis, and meningoencephalitis manifest as inflammation of the brain parenchyma, meninges, or both [1,2]. These conditions can stem from diverse etiologies, including infectious and noninfectious causes [1,2]. A systematic analysis of the Global Burden of Disease (2019) revealed that meningitis caused 236,000 deaths globally in 2019, predominantly affecting children under five years of age, with notable reductions in mortality rates and deaths linked to specific pathogens from 1990 to 2019 [3]. Despite advancements in treatment, the prognosis for meningitis remains unfavorable, with complications such as neurological deficits, hearing impairment, cognitive dysfunction, and epilepsy [1,4].

Globally, viral infections are the primary cause of encephalitis, meningitis, and meningoencephalitis [1]. Notably, herpes simplex viruses, HSV-1 and HSV-2, are commonly associated with encephalitis and meningitis, respectively, as well as enteroviruses [5,6]. Bacterial infections are generally less common than viral infections but carry higher mortality risks with pathogen-specific variations [3]. *Streptococcus pneumoniae* is the leading bacterial pathogen causing meningitis and has the highest mortality among all age groups [3]. Notably, *Klebsiella pneumoniae* has had an increasing prevalence and mortality in the last decade, with increasing antibiotic resistance [3]. Fungal etiologies are rare and often attributed to patients with immune deficiency patients [7]. Parasitic infections leading to encephalitis or meningitis are uncommon but noteworthy, with special clues in presentation [8,9]. However, the frequency and distribution of infectious pathogens vary across populations and geographic regions [3,8].

Diagnosis of meningitis starts with the clinical presentation with a classical triad of fever, altered mentation, neck stiffness, and other physical signs and symptoms [1,8]. The three illnesses have intertwined pathophysiology, clinical course, and complications, which presents a complexity in differentiating the three clinically [8,10]. For instance, multiple protocols have been proposed to diagnose and manage encephalitis [2,9,11-13]. There are numerous mechanisms by which pathogens access and infect the central nervous system, as reviewed by Cain et al. [14]. Investigating the cause requires a cerebrospinal fluid (CSF) analysis, usually acquired via lumper puncture if not contraindicated or extra ventricular drain if present; some pathogens require further modalities, such as brain biopsy, although not done routinely [8]. In addition to culture and real-time reverse transcription-polymerase chain reaction (rRT-PCR), a high-yield test is BioFire® FilmArray® meningitis/encephalitis multiplex panel used to detect specific pathogens in CSF [15]. Imaging modalities of the central nervous system can provide additional diagnostic value by detecting signs of infection or its complications, some of which are pathogen-specific findings [10,16].

Additionally, autoimmune causes of encephalitis, such as anti-N-methyl-D-aspartate receptor (NMDA) encephalitis, are clinically significant causes presenting variable signs and symptoms and often inter-lapping with infectious causes [17].

This study aimed to investigate epidemiological factors and causes linked to meningitis, encephalitis, and meningoencephalitis in a Saudi Arabian population. This retrospective analysis sought to add to medical literature and epidemiological data on central nervous system diseases.

## Materials and methods

The study's design adhered to the Strengthening the Reporting of Observational Studies in Epidemiology (STROBE) guidelines. This retrospective cross-sectional study focused on the population of King Abdulaziz Medical City (KAMC) in Jeddah, Saudi Arabia. The hospital is a tertiary center with a 751-bed capacity. The study included all patients admitted to the KAMC between April 2016 and December 2022 who met the inclusion criteria by screening their medical records.

Inclusion criteria encompassed individuals of all ages and genders diagnosed with encephalitis, meningitis, or meningoencephalitis. Only residents and citizens of Saudi Arabia were eligible for inclusion in this study to minimize geographical variations. The patient population included but was not limited to trauma, oncology, and neurosurgery. All patients were seen and managed by a neurologist, neurosurgeon, intensivist, or infectious disease specialist.

Diagnosis was made based on the results of the CSF sample analysis and culture with samples obtained before initiating empirical treatment. MRI modality was favorable in distinguishing meningitis, encephalitis, and meningoencephalitis. In cases where a lumbar puncture (LP) was contraindicated or not performed, the blood culture and presentation needed to be indicative of meningitis, encephalitis, or meningoencephalitis, supported by imaging (CT or MRI). COVID-19-positive patients were excluded from the study.

Laboratory diagnostic procedures of CSF samples were tested for PCR, cultures, and cell count/differential. For PCR, BioFire® FilmArray® meningitis/encephalitis multiplex panel was used in addition to other pathogens not included in the panel. The panel includes the following viruses: enterovirus, herpes simplex virus type 1 and 2 (HSV-1/HSV-2), varicella-zoster virus (VZV), cytomegalovirus (CMV), human herpesvirus 6 (HHV-6), and human parechovirus (HPeV). The panel includes the following bacteria: *Escherichia coli* K1, *Haemophilus influenzae*, *Listeria monocytogenes*, *Neisseria meningitidis*, *Streptococcus agalactiae*, and *Streptococcus pneumoniae*. The panel includes the fungi *Cryptococcus neoformans* and *Cryptococcus gattii*. Cases diagnosed and identified with autoimmune encephalitis were collected.

Data collection

The data collection sheet recorded information on age, gender, past medical history, presenting signs and symptoms upon admission and discharge, diagnosis, causative pathogen, imaging modality, presence of antibodies, and cause of mortality if deceased.

Statistical analysis

Statistical analyses were performed using IBM SPSS version 26 (IBM SPSS Statistics for Windows, IBM Corp., Armonk, NY). Descriptive statistics, including frequencies, percentages, means, and standard deviations, were used to summarize the categorical and quantitative variables. Significant associations between risk factors and dependent variables were determined using chi-square tests and Fisher’s exact test, with a p-value of <0.05, indicating statistical significance. Multiple regression models were employed to analyze the relationships between variables, and the reliability and validity of the measurements were assessed using Cronbach’s alpha test. Additionally, univariate analysis was conducted to examine the individual relationships between each independent variable and outcome variables. The primary outcome variable under investigation was mortality, while another variable of interest was the GCS score, which was considered as a predictor variable in the analysis. This comprehensive approach facilitated the exploration of relationships and ensured robust analysis of the dataset.

## Results

Diagnosis was initiated by clinical suspicion, followed by CSF samples via LP or external ventricular drain (EVD) when appropriate. The patient’s history and physical examination led the laboratory investigations for specific workups such as *Mycobacterium tuberculosis* and/or adjunct serological workups for pathogen-specific antibodies. Fungal, parasitic, and protozoal cultures were ordered if indicated.

During the period from April 2016 to December 2022, searching the records identified 233 patients at KAMC-J diagnosed with meningitis, encephalitis, or meningoencephalitis. One hundred forty-eight cases of meningitis (65.77%), 23 cases of encephalitis (10.22%), and 54 cases of meningoencephalitis (24%) were identified. Furthermore, eight patients were unable to specify which one of the three categories was due to overlapping underlying conditions such as CNS malignancy. Among the patients, 123 were males (52.8%) and 109 were females (47.8%), with ages ranging from one day to 95 years (Table 1).

**Table 1:** Baseline characteristics of the included population GCS: Glasgow Coma Scale

Baseline characteristics	n = 233	Percentage
Gender		
Male	123	52.8%
Female	109	46.8%
Age	22 (7-57)	
GCS at admission	13(3.1)	
GCS at discharge	12(4.2)	
Mortality		
Alive	189	81.1%
Dead	43	18.4%
Diagnosis		
Meningitis	149	63.9%
Meningoencephalitis	51	21.9%
Encephalitis	26	11.1%
Radiological findings		
Abnormal brain MRI	53	22.8%
Abnormal head CT	32	13.7%
Past medical history		
Hypertension	51	21.8%
Diabetes mellitus	44	18.9%
Cancer	20	8.6%
Previous cerebrovascular disease	18	7.7%
Dyslipidemia	19	8.1%
Known seizure	51	21.9%

The most common chronic diseases were hypertension (21.8%) and diabetes mellitus (18.9%) (Table 1). The presenting signs and symptoms were fever (65.5%), seizures (14.62%), headache (11.5%), and other less common symptoms. One hundred fifty-eight patients had positive culture or multiplex/PCR results. Table 2 presents the distribution of pathogens, with bacterial agents being the most common (79.74%).

**Table 2:** Distribution of causative pathogens NMDA: N-methyl-D-aspartate

Bacterial infection (n = 126, 79.74%)	Frequency (%)
Klebsiella pneumoniae	15 (11.90)
Escherichia coli	14 (11.11)
Staphylococcus aureus	13 (10.31)
*Salmonella *spp.	11 (8.73)
Streptococcus pneumoniae	11 (8.72)
*Shigella *spp.	9 (7.14)
*Campylobacter *spp.	7 (5.55)
Pseudomonas aeruginosa	6 (4.75)
Streptococcus agalactiae	6 (4.75)
Acinetobacter baumannii	5 (3.96)
Mycobacterium tuberculosis	4 (3.17)
Haemophilus influenzae	4 (3.17%)
Staphylococcus epidermidis	4 (3.17%)
*Brucella *spp.	3 (2.38%)
Micrococcus luteus	3 (2.37%)
Enterobacter aerogenes	2 (1.58%)
Others	9 (5.70%)
Viral infection (n = 21, 13.29%)	Frequency (%)
Herpes simplex virus	9 (42.85%)
Enterovirus	3 (14.28%)
Dengue virus	2 (9.52%)
Varicella zoster virus	2 (9.52%)
Cytomegalovirus	1 (4.76%)
West Nile virus	1 (4.76%)
Unknown virus	3 (14.28%)
Fungal infection (n = 6, 3.79%)	Frequency (%)
*Candida *spp.	6 (100%)
Auto-immune CNS Infection (5, 3.16%)	Frequency (%
Anti-NMDA	5 (100%)

The remaining 65 cases fulfilled the clinical presentation of meningeal characteristics but without identifiable pathogen for reasons such as sterile CSF results or contraindications for CSF acquisition. Moreover, the previous cases improved on the administration of antibiotics or antiviral.

At the end of this study, higher mortality was significant with pediatrics <5 years and adults >60 years (p = 0.001). Gender did not have a statistically significant relation. Patients who had a lower GCS on presentation had a lower GCS at discharge, hence, poor prognosis (p = 0.04); however, an insignificant relationship was found with the mortality rates (p = 0.091) (Table 3).

**Table 3:** Univariate analysis of the outcomes GCS: Glasgow Coma Scale *p-value ≤ 0.05

	GCS				Mortality		
	Low	Moderate	High	p-value	Deceased	Alive	p-value
Age							
1-18	10	1	24	0.093	10	98	0.001^*^
19-60	16	2	14		15	55	
61 onward	18	2	11		18	35	
Gender							
Male	25	2	23	0.731	26	97	0.512
Female	19	3	25		17	91	
GCS at admission							
Low	6	2	2	0.004^*^	6	7	0.091
Moderate	5	2	8		5	16	
High	17	-	36		15	65	
Diagnosis							
Meningitis	25	3	29	0.692	23	125	0.030^*^
Meningoencephalitis	14	2	11		15	36	
Encephalitis	3	0	6		2	24	
Presentation							
Temperature > 38°C	40	4	40	0.536	41	159	0.092
Seizures	38	3	35	0.142	38	148	0.135
Headache	12	1	14	0.918	11	53	0.840
Photophobia	38	5	43	0.678	38	165	0.848
Neck stiffness	39	5	47	0.323	39	174	0.768

Different signs and symptoms, including classical triad, did not show a significant relationship with either of the two outcomes, namely, the GCS score at discharge and the mortality rate.

## Discussion

Meningitis and encephalitis, though distinct clinical entities present a substantial challenge in the healthcare industry owing to numerous potential causes [4]. Despite their unique manifestations, they exhibit significant clinical and etiological interconnections. The identification of causative agents is crucial, as many are responsive to medical interventions. However, in over half of the cases, the pathogens causing these conditions remain unidentified, necessitating empirical therapeutic intervention to preempt further clinical decline [18,19]. Patient improvement by treatment provided without a positive culture is a common issue recognized by the guidelines due to situational circumstances [20]. Gaining insight into the demographic frequency and distribution of these etiologies is crucial for effective management and prevention strategies.

In our study, we aimed for epidemiological generalized data by including adult and pediatric populations, not excluding patients with malignancy or neurosurgical cases, and combining all three entities from our local data to add to the epidemiology of these entities. The reported comorbidities align with the current literature, acknowledging the impact of comorbidities on the susceptibility and severity of infectious diseases [18]. Lucas et al. noted that individuals with community-acquired bacterial meningitis rarely present with a minimal GCS score yet are at an elevated risk of morbidity and mortality [21].

Classically, the three meningococcal, pneumococcal, and *Haemophilus influenzae* meningitis were reported as most common in literature. Moreover, studying different regions around the world yields variable results regarding the most common pathogens [3]. In the Middle East and North Africa (MENA) region, however, data are scarce compared to other regions. Concerning Saudi Arabia, a study from Saudi Arabia reported that the most common bacterial causes of meningitis in the pediatric age group are *Haemophilus influenzae* and *Streptococcus pneumoniae* [22]; another cohort observed an incidence peak of *Neisseria meningitidis* during the Hajj season [23]. Another national study reported that the most commonly reported organisms are brucellosis, tuberculosis, and salmonellosis, with an increasing trend of meningitis cases during the Hajj season [24]. The previous study reported that bacterial meningitis other than meningococcal, pneumococcal, and* Haemophilus influenzae* meningitis are far more common than the aforementioned organisms, with the issue of not specifying the organisms in reports [24]. In addition to our results, *Klebsiella pneumoniae* has an increasing prevalence globally, especially in Asian countries, and with significance in meningitis-related mortality [3]. For instance, Zeinalizadeh et al. found *Klebsiella pneumoniae* to be the most frequently infecting species (47%) in postoperative ICU patients with meningitis and encephalitis [25]. Another study for *Klebsiella pneumoniae* found that the organism was associated with multiple virulence factors [26]. Regarding regional data on viral meningitis, one study reported a predominance of enterovirus meningitis among its selected cohort [27]; however, the previous study only studied organisms covered by BioFire® FilmArray® meningitis/encephalitis multiplex panel [27]. Our fungal cases are attributed to *Candida *spp. solely opposite to the globally common pathogen cryptococcus [28]. An epidemiological study in the MENA region did not report *Candida *meningitis cases and reported few cryptococcal cases [29]. The gap in the literature undermines the need for further and more comprehensible epidemiological studies to form knowledge of the most common pathogens and their trends.

Tailoring diagnostic and treatment approaches based on the prevalence of age-specific pathogens can improve patient outcomes. However, identifying the most common causative agent within each age bracket is challenging due to the substantial variations observed, warranting further research to determine if similar differences exist in Saudi Arabia. Moreover, geographical location, income, and healthcare systems are major factors in decreasing meningitis burden [3,4]. Overall, the study provides valuable insights, although limitations, such as potential selection bias and retrospective design, should be acknowledged.

## Conclusions

In conclusion, our research sheds light on the spectrum of encephalitis, meningitis, and meningoencephalitis in the Saudi Arabian population, revealing the diverse probable causative agents and demographic patterns. Refined clinical practices and public health strategies to effectively manage these conditions are necessary. Further studies covering various regions of Saudi Arabia and other MENA regions are necessary to understand the prevalence of these diseases. Additionally, an inferential study is warranted to establish correlations between patient etiologies, neurological complications, imaging findings, and prognosis.
